# Men’s perceptions of women’s sexual interest: Effects of environmental context, sexual attitudes, and women’s characteristics

**DOI:** 10.1186/s41235-016-0009-4

**Published:** 2016-09-22

**Authors:** Teresa A. Treat, Hannah Hinkel, Jodi R. Smith, Richard J. Viken

**Affiliations:** 1 University of Iowa, Iowa City, IA USA; 2 Indiana University, Bloomington, IN USA

**Keywords:** Sexual perception, Context, Sexual aggression, Affect, Cue utilization

## Abstract

Men’s perceptions of women’s sexual interest were studied in a sample of 250 male undergraduates, who rated 173 full-body photos of women differing in expressed cues of sexual interest, attractiveness, provocativeness of dress, and the social-environmental context into which the woman’s photo had been embedded. Environmental context significantly influenced men’s judgments of sexual interest, independently of the affective cues of sexual interest themselves and of provocativeness of dress and attractiveness. Cue usage was moderated by men’s risk for sexual aggression, as measured by a rape-myth inventory, with higher-risk men (relative to lower-risk men) relying significantly less on affective cues, relying significantly more on attractiveness, and showing a non-significant tendency to rely more on environmental cues. Men exhibited a moderate degree of insight into individual differences in their cue usage. Analysis of individual differences in cue usage suggested that men’s judgments of women’s momentary sexual interest varied along two dimensions: (1) men who relied more on affective cues were less likely to rely on women’s attractiveness (r = −0.73); and (2) men who were influenced more by provocativeness of dress were also likely to rely more on environmental context (r = 0.49). Results suggest that variation in contextual variables should be included in cognitive-training programs designed to improve the accuracy of men’s judgments of women’s affective responses. Ultimately, such training programs may prove useful as an adjunct to prevention programs for sexual aggression.

## Significance

Male-initiated sexual aggression toward female acquaintances is a serious behavioral-health problem on college campuses. Current theories suggest that misperception of the sexual interest of a potential sexual partner may increase the likelihood of sexually coercive and aggressive behavior. The emotional cues that a woman expresses on her face and in her body posture provide nonverbal indicators of how she feels about a particular man at a particular point in time. When judging sexual interest, however, men focus not only on emotional cues but also on the provocativeness of women’s clothing and their attractiveness. Moreover, men at greater risk of exhibiting sexual aggression focus less than their peers on emotional cues and more on women’s attractiveness when judging sexual interest. The current work evaluates whether the social-environmental context in which women appear also influences men’s perceptions of women’s momentary sexual interest. Women were placed in scenes that were either lower in sexual relevance (e.g., sidewalk, class, office reception area) or higher in sexual relevance (e.g., bar, house party, or bedroom). When judging women’s sexual interest, men focused on environmental cues, attractiveness, and provocativeness of clothing independently of expressed emotional cues, and men’s risk for sexual aggression moderated the degree to which they focused on some of these dimensions. Results suggest that variation in environmental context, dress, and attractiveness should be included in cognitive-training programs designed to improve the accuracy of men’s judgments of women’s emotions, which eventually might be used to augment prevention efforts in sexual aggression.

## Background

Sexual perception—that is, perceiving someone’s level of sexual interest in a particular person at a particular point in time—is a common, difficult, and potentially consequential task. Most of the time, a man’s misperception of a woman’s level of sexual interest will be a minor but potentially embarrassing social error, in which he either perceives a woman to be more interested than she actually is or perceives a woman to be less interested than she actually is. More importantly, however, sexual misperception also is associated both theoretically and empirically with an increased likelihood of sexually coercive and aggressive behavior toward acquaintances, whether indexed by a self-reported history of aggression or endorsement of rape-supportive attitudes (for more recent reviews of this extensive empirical literature, see Abbey, Jacques-Tiura, & LeBreton, 2011, and Farris, Treat, Viken, & McFall, 2008a). Standardized social-perception tasks using numerous, well-characterized, full-body photographs of college women allow examination of both nomothetic and idiographic aspects of sexual perception—that is, both average (i.e., nomothetic) sexual perception and individual differences in (i.e., idiographic) sexual perception—under highly controlled conditions. To date, work under these conditions has focused primarily on characteristics of the women being perceived and of the men doing the perceiving when accounting for variability in sexual perception. The current study extends prior research by rigorously evaluating the role of the social-environmental context in sexual perception while controlling for women’s nonverbal affect, the perceived provocativeness of women’s clothing, and women’s normative attractiveness, as well as their interactions.

### Nomothetic aspects of sexual perception

**Fig. 1:**
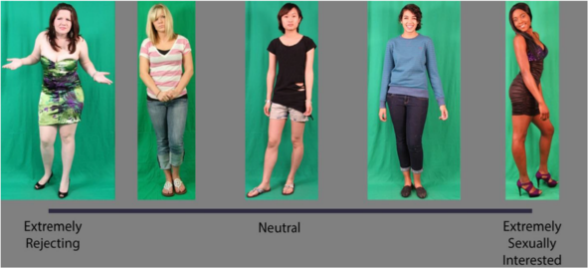
Models whose momentary sexual interest ranges from extremely rejecting (on the left), through neutral (in the middle), to extremely sexually interested (on the right), as judged by both undergraduate women and experts in sexual perception

On average, college men base judgments of a woman’s momentary sexual interest in a full-body photograph on multiple characteristics of the woman, such as her nonverbal affect, the perceived provocativeness of her clothing style, and her normative attractiveness (Treat, Church, & Viken, under review; Treat, Viken, Farris, & Smith, in press). Affective cues presumably are the most valid nonverbal indicators of a woman’s current level of sexual interest in a particular person because they fluctuate from moment to moment and can be unidirectional (i.e., directed at a specific person). Figure 1 depicts five women whose momentary sexual interest ranges from extremely rejecting (on the left), through neutral (in the middle), to extremely sexually interested (on the right), as judged by both undergraduate women and experts in sexual perception (see “Methods” for further detail). Inspection of these photos suggests that nonverbal affect is communicated both on a woman’s face and in her body posture, highlighting the utility and greater ecological validity of relying on full-body photographs rather than head shots when studying sexual perception.

In contrast to affective cues, non-affective cues, such as clothing style and attractiveness, provide far less information about a woman’s momentary level of sexual interest because they typically are quite stable across a social interaction and tend to be more omnidirectional (i.e., available to everyone in the social environment). Not surprisingly, men rely heavily on women’s nonverbal affective cues when judging women’s current sexual interest: two recent studies have documented that men’s sexual-interest judgments showed impressively strong associations with women’s nonverbal affective cues (*d*s = 2.79, 3.35; Treat et al., in press, under review). Note that Cohen’s *d* values indicate the magnitude of the two effects, where values of 0.2, 0.5, and 0.8 are considered to be small, moderate, and large effects in the psychological literature (Cohen, 1992). Non-affective characteristics of women also non-neglibly predict men’s judgments, however, over and above affective characteristics: in the same two studies, men’s sexual-interest judgments were moderately associated with women’s clothing style (*d*s = 0.78 and 0.65) and strongly associated with women’s attractiveness (*d*s = 0.94 and 1.16; Treat et al., in press, under review). In other words, the average participant judged women who were dressed provocatively and were more normatively attractive to be feeling much more sexually interested than other women at a specific moment in time, even when the women’s nonverbal expression of sexual interest was held constant. Particularly the reliance on attractiveness when judging women’s momentary affect may reflect projection of the affect of the male perceiver onto the female being perceived in a manner that is consistent with the perceiver’s interpersonal goals (Maner et al., 2005; Maner, Miller, Moss, Leo, & Plant, 2012). Regardless, it is easy to imagine how reliance on women’s dress and attractiveness when judging women’s fluctuating feelings could set the stage for unwanted advances.

An extensive social psychological literature demonstrates that emotional perception does not occur in a decontextualized manner. Rather, contextual information in the body and in the social environment typically is encoded along with emotion in faces (e.g., Barrett & Kensinger, 2010; de Gelder et al., 2006; Kret & de Gelder, 2010, 2012; Kret, Roelofs, Stekelenburg, & de Gelder, 2013; Van den Stock, Righart, & de Gelder, 2007). Consistent with a “congruence hypothesis”, contexts that are more, rather than less, congruent with particular emotional interpretations facilitate and speed those interpretations (e.g., Aviezer, Hassin, Bentin, & Trope, 2008; Kret & de Gelder, 2010, 2012). This makes it critically important for researchers to examine social and emotional perception in a contextualized manner.

A recent study suggests that the sexual and dating relevance of the socio-environmental context also shape men’s perceptions of women’s sexual interest (Treat, Viken, & Summers, 2015b). Twenty-eight scenes were constructed that depicted social environments that were either lower in sexual relevance (e.g., sidewalk, class, office reception area) or higher in sexual relevance (e.g., bar, house party, or bedroom). A full-body photograph of one of 14 college-aged women was inserted into two scenes that varied in sexual relevance; the women all expressed neutral-to-positive affect and varied in provocativeness of dress and attractiveness. College men viewed each scene and judged how sexually interested and friendly each woman felt on Likert scales. Consistent with a congruence hypothesis, sexually relevant contexts potentiated men’s judgments of women’s sexual interest to a far greater degree than men’s judgments of women’s friendliness (*d*s = 1.75, 0.33, respectively). This suggests that the sexual relevance of the environmental context may be another omnidirectional cue on which college-aged men rely when evaluating how sexually interested women are feeling.

This prior work was limited in several respects, however (Treat, Farris, Viken, & Smith, 2015a). First, participants were asked to make judgments of only a small number of women and scenes. Thus, we were unable to estimate the extent to which each participant relied independently on each of the four stimulus dimensions of primary psychological interest when making his sexual-interest judgments. We also were unable to examine the associations between reliance on each of these dimensions and relevant individual-difference variables, such as endorsement of rape-supportive attitudes. Second, Treat et al. (2015b) focused exclusively on men’s perceptions of women’s non-negative affect (i.e., the most “negative” affect displayed was neutral rather than sad or rejecting). Ideally, however, we would characterize individual differences in men’s reliance on a broader range of affective cues when judging women’s sexual interest, particularly given the relevance of men’s perceptions of women’s negative affect to the initiation and cessation of unwanted sexual advances. Third, Treat et al. (2015b) did not specify the time period over which participants judged women’s sexual interest. It would be preferable to obtain judgments of women’s momentary sexual interest, however, as this would render clothing style, attractiveness, and the social environment far less plausible indicators than the fluctuating nonverbal information on the face and in the body.

The current study extends this prior work by developing and evaluating men’s judgments of a much larger set of unique scenes for which affect, clothing style, attractiveness, and contextual sexual relevance vary independently. Affective variability also ranges from extremely rejecting to extremely sexually interested. This allows us to obtain simultaneous but independent estimates of both woman-specific and context-specific influences on the full spectrum of men’s judgments of women’s sexual interest. We also ask participants to judge how sexually interested the woman is feeling “right now”, which isolates men’s judgments of women’s momentary sexual interest.

Further, we evaluate for the first time in a continuous-rating paradigm whether these four cues influence sexual perception in only an additive fashion (i.e., as main effects only) or potentially in a multiplicative fashion. We have observed such multiplicative effects in much of our prior sexual-perception work in which participants have classified the woman’s affect (e.g., sexually interested, friendly, sad, or rejecting) rather than judging the woman’s affect on a continuous-rating scale, as in the current work. For example, men’s sensitivity to women’s sexual interest is potentiated when women are dressed provocatively or are normatively attractive, whereas men’s sensitivity to women’s rejection is potentiated when women are dressed conservatively or are normatively unattractive (Farris, Treat, Viken, & McFall, 2008b; Farris, Viken, & Treat, 2010; Farris, Viken, Treat, & McFall, 2006; Smith, Treat, Farmer, & McMurray, under review; Treat et al., 2015a). In other words, sensitivity to particular affective cues is enhanced when more congruent levels of non-affective cues are present, consistent with the congruence hypothesis cited above regarding contextual influences on emotion perceptions (Aviezer et al., 2008; Kret & de Gelder, 2010, 2012). In the context of the current continuous-rating paradigm, these robust findings suggest that reliance on affect might be potentiated by provocative clothing style, attractiveness, and sexually relevant contexts.

### Idiographic aspects of sexual perception

Marked individual differences in the basis of men’s judgments about women’s momentary sexual interest also emerge across studies. Consistent with contemporary models of sexual aggression between acquaintances (Abbey et al., 2011), men who focus less than their peers on women’s nonverbal affective cues when judging women’s momentary sexual interest endorse more rape-supportive attitudes, placing them at greater risk of exhibiting sexually coercive or aggressive behavior (*d*s = −0.43, −0.36, respectively; Treat et al., in press, under review). Moreover, men who focus more than their peers on women’s attractiveness when judging how sexually interested women feel also are at greater risk (*d*s = 0.32, 0.40, respectively; Treat et al., in press, under review), raising the possibility that higher-risk men are more likely than lower-risk men to conflate their own affective response to a woman with the woman’s expressed affect (Maner et al., 2005, 2012). Given these findings, it is perhaps unsurprising that high-risk men report misperceiving women’s friendliness as sexual interest more than their peers (e.g., Abbey, 1987; Jacques-Tiura, Abbey, Parkhill, & Zawacki, 2007). Interestingly, individual differences in the influence of sexually relevant contexts on men’s sexual-interest judgments correlated positively with the reported frequency of misperception experiences (Treat et al., 2015b). Overall, therefore, variability in the basis of men’s sexual-perception judgments may be associated with risk for coercive or aggressive behavior, perhaps secondary to misperception of sexual-consent cues or to frustration in response to a woman’s seemingly capricious change in her level of sexual interest (Abbey et al., 2011; Farris et al., 2008b).

The current study advances our understanding of idiographic aspects of sexual perception by evaluating the relation of rape-supportive attitudes, a well-established correlate of sexually aggressive behavior (Murnen, Wright, & Kaluzny, 2002), with reliance on affect, clothing style, attractiveness, and contextual sexual relevance when judging women’s momentary sexual interest. This affords a first look at whether those at greater risk of displaying sexually aggressive behavior focus more than their peers on contextual sexual relevance, as they do attractiveness (Treat et al., in press, under review). We also obtain estimates of the attitudinal links with reliance on the other three woman-specific dimensions when using more ecologically valid scene stimuli. Furthermore, we evaluate for the first time the bivariate (pairwise) correlations of cue-utilization values across participants (i.e., the six associations between all possible pairs of the four cue-utilization values). Pairwise correlations between reliance on context and each of the three woman-specific dimensions tell us whether reliance on each cue is relatively independent of reliance on the other cues or whether there are meaningful relations between reliance on two or more cues. For example, men who rely relatively more on affective information when judging women’s sexual interest might rely less on attractiveness information and vice versa. Ultimately, such analyses may provide a useful perspective on the underlying structure of men’s judgments of women’s nonverbal dating-relevant cues.

Finally, the current study characterizes men’s self-reported cue-utilization patterns. The association between self-reported and observed cue utilization tells us about the extent to which the average participant is aware of the cues on which he relies when judging women’s momentary sexual interest. Non-negligible awareness would suggest that cue-utilization patterns might be more responsive to deliberate efforts on the part of participants to modify them, perhaps secondary to explicit instruction about the relative validity of the cues for determining women’s momentary affect (Treat et al., under review). Male participants in a prior study reported their use of affect, clothing style, and attractiveness cues after completing the sexual-interest judgment task. Moderate-to-large associations with observed cue utilizations emerged (*r*s = 0.47, 0.25, 0.39, respectively; Treat et al., under review). The current study extends this work by obtaining self-reported estimates of focus on contextual sexual relevance in addition to the other three dimensions. We also examine the association of rape-supportive attitudes with self-reported cue utilizations so that we can determine whether higher-risk men are aware of their presumably decreased focus on affect and increased focus on attractiveness when judging women’s momentary affect.

In sum, the current work examines the hypotheses that environmental context, like the non-affective cues of dress and attractiveness, would affect men’s judgments of women’s sexual interest; that patterns of cue usage would be moderated by risk for sexual aggression; that utilization of affective cues would be moderated by non-affective cues present in the stimuli; and that men would have some degree of awareness of their patterns of cue usage.

## Methods

### Participants

Participants were 270 male undergraduates who received partial course credit for completing the study. Twenty participants were dropped from all analyses: six were not between the ages of 18 and 24 years, three were married, three were not heterosexual or bisexual, three were missing more than 10 % of their Illinois Rape Myth Acceptance (IRMA) data, and five showed extremely minimal variability in rating data. The final sample contained 250 unmarried, heterosexual or bisexual, male undergraduates between the ages of 18 and 24 years. The average age of participants was 19.32 years (standard deviation (SD) = 1.35); 70.8 % identified as Caucasian/White, 10.4 % as Asian-American/Asian, 5.6 % as African-American/Black, and 6.4 % as Mexican-American; 93.2 % reported at least one serious or casual dating relationship in the last 3 years. 

### Scene stimulus set

Stimuli were 173 full-body photographs of unique undergraduate females integrated into real-world scenes. The women’s photos were selected from a set of 3129 photos of women that were taken by the research team (see also Treat et al., under review). The models wore warm-weather clothing that varied in sexual provocativeness and they were asked to display a wide variety of affective cues across photographs, including rejection, friendliness, neutrality, sexual interest, and sadness. Thus, the women in the stimululs set varied along three psychological dimensions: sexual interest (extremely rejecting to extremely sexually interested), provocativeness of dress (conservative to provocative), and normative attractiveness. Nine undergraduate women provided normative ratings of the models’ sexual interest and provocativeness-of-dress. When judging sexual interest, raters were instructed “to focus only on the degree to which the woman is expressing sexual interest, and to ignore her clothing style, her attractiveness, your personal reactions to the woman or her clothing, etc.” When judging provocativeness of dress, raters were instructed to 'focus only on the sexual provocativeness of the clothing and to ignore characteristics of the model completely, including her affect, her attractiveness, how she looks in the clothing, your personal reactions to the woman or her clothing, etc.’ Thus provocativeness of dress throughout this manuscript refers to a subjective judgment about the woman's clothing by relevant raters, rather than to an inherent property of the woman or her clothing. Inter-rater reliability among the coders was high (intraclass correlation coefficient (ICC) = 0.97, 0.95). Members of our larger research team with expertise in sexual perception also coded sexual interest and provocativeness of dress. The average ratings of undergraduate women and experts converged strongly (r = 0.97, 0.96, respectively). A large sample of undergraduate men rated the women’s attractiveness on a ten-point scale.

**Fig. 2:**
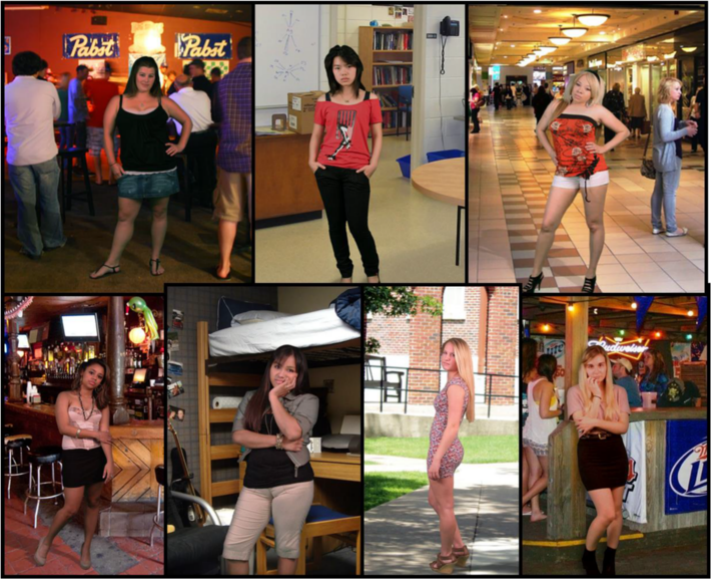
Sample scenes presented in the sexual-interest judgment task that vary along four psychological dimensions of interest: woman’s sexual interest, provocativeness of dress, and normative attractiveness, as well as sexual relevance of context

The scenes into which the women were integrated were selected for this study from a set of 150 scenes in which college men and women might interact that were obtained from the internet. The same group of nine undergraduate women judged the relevance of each scene to sexual activity or the pursuit of sexual activity (sexual relevance) on a seven-point scale. We selected 108 scenes for use in this study if they received sexual relevance ratings < 3 or > 5. No scene was used more than three times when creating the final set of 173 scenes that the participants viewed. Of the final scenes, 87 (50.3 %) were high in sexual relevance (e.g., bar, bedroom) and 86 (49.7 %) were low in sexual relevance (e.g., office, store). Figure 2 depicts sample scenes.

The normative ratings from the undergraduate women and men were used to compute an average value for each scene along each of the four dimensions. To minimize multicollinearity, correlations between normative ratings for the four dimensions were minimized during stimulus selection (all *r*s < |0.15|). This made it possible to obtain independent estimates of reliance on sexual interest, provocativeness of clothing style, attractiveness, and the sexual relevance of the context from participants’ judgments on the sexual-interest judgment task.

### Tasks and measures

#### Sexual-interest judgment task

**Fig. 3:**
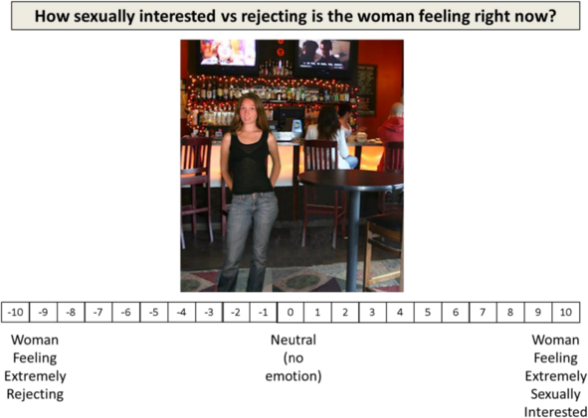
Sample trial in the sexual-interest judgment task

Participants viewed 173 scenes for 2 s apiece in a random order. Participants were told that they would be “judging how sexually interested versus rejecting 173 women feel right now,” using “a scale that ranges from −10 = extremely rejecting, to 0 = neutral, to 10 = extremely sexually interested.” Figure 3 presents a sample trial.

#### Self-reported cue utilization

Participants answered four questions about their utilization of attractiveness, clothing provocativeness, nonverbal emotional cues, and contextual sexual relevance when judging women’s sexual interest: “In the task you just completed, how much do you think your judgments of women’s sexual interest were influenced by the woman’s attractiveness / the provocativeness of the woman’s clothing/the woman’s nonverbal emotional cues (e.g., facial expression, body posture) / the relevance of the background scene to dating or sexual activity?” The response scale ranged from 0 (“not at all influenced by this characteristic”) to 100 (“extremely influenced by this characteristic”).

#### Illinois Rape Myth Acceptance

The IRMA (Payne, Lonsway, & Fitzgerald, 1999) is a 45-item questionnaire that assesses endorsement of rape-supportive attitudes. Participants responded on a seven-point scale (1 = “not at all agree”, 7 = “very much agree”). Sample items include “Men don’t usually intend to force sex on a woman, but sometimes they get too sexually carried away”, “If a woman is raped while she is drunk, she is at least somewhat responsible for letting things get out of control”, and “When women go around wearing low-cut tops or short skirts, they’re just asking for trouble.” The average score on the IRMA was 2.52 (SD = 0.76).

#### Personal information questionnaire

Participants reported demographic characteristics (i.e., age, race/ethnicity, marital status, sexual orientation), as well as dating history (i.e., number of casual and serious dating relationships over the last 3 years).

### Procedure

After completing an informed-consent statement, each participant was seated in a private booth in front of a computer. He then completed the tasks and measures in the order described above.

## Results

### Sexual-interest judgment task

A mixed-effects model was fit to participants’ sexual-interest judgments using the lmer function in the linear mixed-effects package lme4 (Bates, Maechler, Bolker, & Walker, 2015) in R (R Core Team, 2016). The mixed-effects analytic approach allowed us to estimate simultaneously but separately the main effects of scene-specific characteristics (e.g., dress, attractiveness, contextual sexual relevance) and participant-specific characteristics (e.g., IRMA) on men’s judgments of women’s sexual interest, as well as any interactive effects between scene-specific and participant-specific characteristics on men’s judgments. For example, the main effect of women’s attractiveness on men’s judgments of women’s sexual interest would indicate whether men relied significantly on women’s attractiveness when judging women’s sexual interest, and an associated effect-size value (Cohen’s *d*) would indicate the magnitude of such reliance or cue utilization, relative to no utilization of that cue. As another example, an interactive effect of women’s attractiveness and men’s IRMA would indicate whether men who endorsed more rape-supportive attitudes relied on women’s attractiveness to a greater degree than their peers when judging women’s momentary sexual interest. Here, too, an associated effect size would indicate the magnitude of the moderation of the main effect of attractiveness by men’s IRMA. Bivariate interactions between scene characteristics were included but were not moderated by IRMA to simplify the model.

Degrees of freedom and *p* values for all main effects and interactions were obtained from the lmerTest package (Kuznetsova, Brockhoff, & Christensen, 2013). All continuous predictors were centered for analysis (IRMA and woman-specific characteristics) and contextual sexual relevance was effect coded (1 = high sexual relevance, −1 = low sexual relevance).

**Fig. 4:**
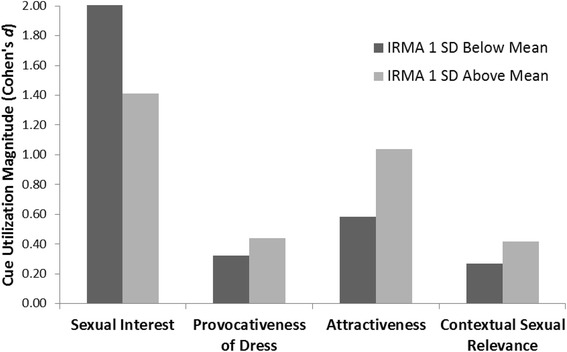
Model-predicted utilization magnitude (Cohen’s *d*) for four scene characteristics for those with IRMA scores 1 SD below and above the mean. *IRMA* = Illinois Rape Myth Acceptance

The random-effects structure included the following components: 1) random intercepts for both subject and scene (i.e., average sexual-interest judgments of the average woman were allowed to vary across participants and across scenes); 2) random subject slopes for woman- and scene-specific characteristics (i.e., the magnitude of the association between these characteristics and men’s sexual-interest judgments was allowed to vary across participants); and 3) random covariances between the random subject intercepts and slopes (i.e., all pairwise associations among the random intercepts and slopes were allowed to vary across participants). The specification of fixed effects and random effects in lmer was as follows: lmer(Sexual-interest rating ~ IRMA * (Sexual interest + Provocativeness of dress + Attractiveness + Contextual sexual relevance) + Sexual interest * (Provocativeness of dress + Attractiveness + Contextual sexual relevance) + Provocativeness of dress * (Attractiveness + Contextual sexual relevance) + Attractiveness * Contextual sexual relevance + (1 + Sexual interest + Provocativeness of dress + Attractiveness + Contextual sexual relevance | subject) + (1 | scene)). We present all reliable and trend-level nonsignificant findings with two-tailed *p* values below. Note that Fig. 4 presents the model-predicted utilization magnitude (Cohen’s *d*) for the four scene characteristics for those with IRMA scores 1 SD above and below the mean.

#### Summary of findings

As detailed below, college men on average relied on all four psychological dimensions of the scenes when judging women’s sexual interest; greater endorsement of rape-supportive attitudes was associated with lesser reliance on women’s affect and greater reliance on women’s attractiveness; and men’s reliance on women’s affect and attractiveness showed a very strong negative association.

#### Sexual-interest utilization effects

Participants relied very strongly on women’s affective cues when judging how sexually interested they feel: *B* = 0.493, *t*(392.6) = 20.660, *p* < 0.001, *d* = 2.09. Endorsement of rape-supportive attitudes predicted moderately reduced reliance on affect: *B* = −0.003, *t*(248.0) = −4.408, *p* < 0.001, *d* = −0.56. Thus, higher-risk men focused less than their peers on women’s momentary affect when judging how sexually interested women feel “right now”.

#### Provocativeness-of-dress utilization effects

Participants relied moderately on women’s clothing style when judging their momentary sexual interest: *B* = 0.142, *t*(224.7) = 3.574, *p* < 0.001, *d* = 0.48. Endorsement of rape-supportive attitudes was unrelated to utilization of clothing style: *B* = 0.001, *t*(248.0) = 1.205, ns, *d* = 0.15.

#### Attractivenesss utilization effects

Participants relied strongly on women’s attractiveness when judging their sexual interest: *B* = 0.649, *t*(394.1) = 9.884, *p* < 0.001, *d* = 1.00. Rape-supportive attitudes positively predicted moderate-magnitude reliance on attractiveness: *B* = 0.006, *t*(248.0) = 3.625, *p* < 0.001, *d* = 0.46. In other words, men at greater risk of exhibiting sexually coercive and aggressive behavior, relative to their peers, focused more on women’s physical characteristics when judging their current level of sexual interest.

#### Sexual relevance utilization effects

Participants relied moderately on the sexual relevance of the context when judging women’s sexual interest: *B* = 0.200, *t*(192.8) = 2.790, *p* < 0.01, *d* = 0.40. Endorsement of rape-supportive attitudes was related weakly, and only at a trend level, to utilization of contextual sexual relevance: *B* = 0.001, *t*(248.1) = 1.712, *p* < 0.10, *d* = 0.22. To a small and non-significant degree, therefore, higher-risk men relied more than lower-risk men on the sexual relevance of the context when judging how sexually interested women feel.

#### Intercept effects

The average sexual-interest judgment was 0.161, which did not differ reliably from 0.0, the middle of the scale, *t*(397.2) = 1.475, ns, *d* = 0.15. Endorsement of rape-supportive attitudes was unrelated to average judgments: *B* = 0.0005, *t*(248.0) = 0.196, ns, *d* = 0.03. 

#### Bivariate scene-characteristic interactions

All pairwise interactions between the four scene characteristics were unreliable and small in magnitude: all *p* values >0.25, all *d*s < 0.20. Thus, for example, the extent to which participants in general focused on women’s affective cues did not increase in the presence of higher levels of non-affective cues, such as provocativeness of dress or sexually relevant contexts.

#### Bivariate correlations among cue utilizations for four dimensions

The bivariate correlations among cue-utilization estimates for the four dimensions were estimated based on the variance-covariance matrix for subject-specific random intercepts and slopes. Note that Cohen (1992) recommended that correlations of 0.1, 0.3, and 0.5 be described as small, medium, and large, respectively, in the psychological literature. Reliance on affective and attractiveness information showed a very strong negative association, *r* = −0.73. Thus, participants who relied heavily on affect relied minimally on attractiveness and vice versa. Reliance on clothing style and contextual sexual relevance showed a strong positive correlation, *r* = 0.49, indicating that participants who relied on clothing style also tended to rely on context when judging women’s sexual interest. All other pairwise associations were less than |0.25|.

### Self-reported cue utilization

Participants reported relying most on women’s nonverbal emotional cues, *M* = 81.65 (SD = 20.59), second-most on women’s attractiveness, *M* = 61.14 (SD = 28.26), and less on both clothing style and contextual sexual relevance, *M*s = 54.81 (SD = 26.45) and 48.55 (SD = 28.17). Endorsement of rape-supportive attitudes showed a weak negative association with self-reported reliance on sexual interest, *r*(245) = −0.196, *p* < 0.01, and a weak positive association with self-reported reliance on provocativeness-of-dress and attractiveness, *r*s(245,246) = 0.217 and 0.228, *p* values <0.01. Self-reported and observed cue utilization correlated moderately to strongly for three cues—nonverbal emotional cues, *r*(245) = 0.557, *p* < 0.001, provocativeness of clothing, *r*(245) = 0.342, *p* < 0.001, and attractiveness, *r*(246) = 0.544, *p* < 0.001—and weakly to moderately for contextual sexual relevance, *r*(246) = 0.208, *p* < 0.001.

## Discussion

Misperceiving a potential partner’s level of sexual interest can have important social consequences. Most concerning, and consistent with current theoretical models and empirical data, misperception of sexual interest may increase risk of sexually coercive and aggressive behavior (e.g., Abbey et al., 2011; Farris et al., 2008b). In the “real world”, men’s judgments of women’s sexual interest might be informed by dynamic and potentially interacting characteristics of the male perceivers (e.g., their goals, attitudes, and physiological states), the women being perceived (e.g., their verbal and nonverbal cues, their clothing, their physical attractiveness), and the social environment (e.g., its relevance to sexual and dating activity, the presence of alcohol or drugs, the input of peers). Fortuitously, we can obtain an illuminating window onto both nomothetic and idiographic aspects of this phenomenon from observing men’s performance on a standardized and highly simplified laboratory task, in which men judge the momentary sexual interest of a large number of well-characterized women depicted in full-body photos. The current work significantly extends much of our prior work (Treat et al., in press, under review) by carefully embedding these women into a large number of well-characterized scenes that vary in their relevance to dating and sexual activity. This enhances the ecological validity of the stimulus set, which is desirable because men do not judge decontextualized women in the “real world”. Simultaneously, we improve upon the internal validity of our prior study in which men judged the sexual interest of women depicted in scenes (Treat et al., 2015b), so that we obtain rigorous independent estimates of participants’ utilization of all four stimulus dimensions. Overall, therefore, examination of participant judgments of these carefully developed and selected “thin slices” of women’s communication of dating-relevant cues in socially relevant contexts (Ambady & Rosenthal, 1992) allows us to draw more externally valid inferences with greater confidence about both nomothetic and idiographic aspects of sexual perception.

### Nomothetic findings

Consistent with the findings of two prior studies using the same judgment task but presenting decontextualized women (Treat et al., in press, under review), the current study demonstrates that, on average, college men who judge women’s momentary sexual interest rely very strongly on women’s affective information, strongly on women’s normative attractiveness, and moderately on the perceived provocativeness of women’s clothing (*d*s = 2.09, 1.00, 0.48). Because affect, clothing style, and attractiveness vary independently in the presented stimulus set, we can conclude that men’s judgments of a woman’s current sexual interest, on average, increase non-negligibly when the woman is normatively attractive and wears more provocative clothing, even when her current affective expression of sexual interest is held constant. This could decrease the likelihood that men detect attractive and provocatively dressed women’s changing levels of sexual interest, including sexual disinterest and declining sexual interest, which could set the stage for unwanted sexual advances.

In two prior studies using this paradigm, we evaluated the impact of simple experimental manipulations on cue utilization. First, we examined the effect of trial-by-trial feedback on men’s sexual-interest judgments (Treat et al., in press). After judging a woman’s sexual interest on each trial, half of the participants viewed the average judgment of the woman’s sexual interest by sexual-perception experts. Second, we evaluated the impact of explicit instruction about the differential validity of affective and non-affective indicators of women’s momentary sexual interest prior to completion of the judgment task. In this case, half of the participants viewed didactic information about nonverbal indicators of women’s sexual interest before making their judgments. We found that both approaches reduced reliance on omni-directional cues, like dress and attractiveness, and increased reliance on unidirectional affective cues. Although these effects were observed only in a laboratory context, they suggest the potential utility of developing and evaluating more comprehensive cognitive-training programs in this area (Treat et al., in press, under review).

Our prior work suggested that the sexual relevance of the socio-environmental context might function as an additional cue on which college men rely when judging women’s sexual interest (Treat et al., 2015b). However, the strength and generalizability of the inferences drawn from this pilot work were constrained by presentation of a small number of scenes that did not vary independently along all four dimensions of theoretical interest and by inclusion of women displaying only neutral and positive affect. The current work, in contrast, clearly establishes that college men, on average, indeed do rely independently on contextual sexual relevance as a third omni-directional indicator of women’s momentary sexual interest (*d* = 0.40), at a moderate magnitude similar to that of clothing style (*d* = 0.48). This finding underscores what emotion scientists have been demonstrating with respect to judgments of the more “classic” affective states of others, such as happiness, sadness, anger, and fear—namely, that such judgments are influenced not only by what is going on “below the neck” (e.g., in a person’s body language) but also by the context external to the body (e.g., de Gelder et al., 2006). Much like a celebratory context might enhance detection of affectively congruent states such as happiness and reduce detection of affectively incongruent states such as sadness (e.g., de Gelder et al., 2006), sexually relevant contexts might potentiate judgments of sexual interest and inhibit judgments of sexual rejection.

Sexually relevant contexts, by definition, are associated with dating and sexual activity, but a woman’s level of sexual interest within these contexts may fluctuate over time and vary as a function of the man with whom she is interacting. Thus, learning to dissociate judgments of a woman’s momentary sexual interest from the context in which she appears could decrease the likelihood that a man makes unwanted sexual advances, whether the woman is a new acquaintance or an established partner. This suggests the potential utility of implementing training strategies, such as trial-by-trial feedback and explicit instruction (Treat et al., in press, under review), with women presented in scenes that vary in sexual relevance rather than in decontextualized full-body photographs. Presumably the incorporation of environmental context into the training stimuli would slow men’s initial learning about women’s momentary affect, as participants on average would have to learn to focus less not only on clothing style and attractiveness but also on the sexual relevance of the context. The greater ecological validity of the stimuli, and the inclusion of another “distractor” dimension in the stimuli, however, might enhance the robustness of what is learned and facilitate transfer to more “real-world” situations.

Intentional selection of scenes with minimal pairwise associations among the four stimulus dimensions afforded the opportunity to obtain simultaneous but independent estimates of men’s average utilization of these four cues when judging women’s sexual interest. Our prior work with affective classification tasks (rather than continuous-rating tasks) revealed interactive effects of affective and non-affective cues based on their congruence: men showed greater sensitivity to a sexual-interest cue when women were provocatively dressed or attractive and greater sensitivity to a rejection cue when women were conservatively dressed or unattractive (Farris et al., 2006, 2008b, 2010; Smith et al., under review; Treat et al., 2015a). The extent to which pairwise cue combinations influence men’s continuous sexual-interest judgments over and above the independent contributions of the cues to such judgments has been unknown, however. Augmenting our main-effects model (e.g., main effects for each of the four dimensions on men’s judgments) by including the six bivariate interactions among the four dimensions allowed us to investigate this possibility in the current study.

Contrary to expectations and our prior work (e.g., Farris et al., 2006, 2008b, 2010; Treat et al., 2015a), all six bivariate interactions among the cues were unreliable and small in magnitude. The lack of interactions between utilization of affect and the other cues indicated that provocative dress, attractiveness, and sexually relevant contexts did not potentiate reliance on affect when judging women’s sexual interest on a continuous rating scale. These null findings are contrary to the congruence effects that we have observed in our prior categorization work, in which participants classified women’s displayed affect into sexual-interest, rejection, friendliness, or sadness categories (e.g., men showed greater sensitivity to women’s sexual interest when the women dressed provocatively rather than conservatively). Notably, however, congruence effects in this prior work emerged for some affective categories (sexual-interest, rejection, and sadness categories) and not for others (friendliness). Thus, our reliance on a classification task in prior studies examining the congruence hypothesis allowed us to estimate the effects of clothing style separately for each affective cue, such that we could observe effects on only a subset of the affective cues (e.g., not for friendly affect). In contrast, our reliance on a continuous rating scale in the current study necessitated estimation of the effects of non-affective cues across a broad range of affect, even though sensitivity to some affective cues appeared in prior work to be unrelated to values of non-affective cues. Thus, future investigations of congruence-related hypotheses may benefit from relying on variants of the classification task, which appears to be more sensitive to congruence effects. In particular, more formal methods for evaluating perceptual and decisional independence of cues (e.g., General Recognition Theory; Ashby & Townsend, 1986; Farris et al., 2010) might prove valuable, although they would require different tasks and analytic strategies than those used in our standard classification task.

### Idiographic findings

Men’s misperception of a potential female partner’s sexual interest is not only a normative phenomenon affecting a majority of men but also a potentially clinically relevant phenomenon. Misperception is associated both theoretically and empirically among men with an increased risk of exhibiting sexually coercive and aggressive behavior, as commonly indicated by endorsement of rape-supportive attitudes (e.g., Murnen et al., 2002). Consistent with the findings observed in the two prior studies using the same continuous-rating judgment paradigm but decontextualized women (Treat et al., in press, under review), rape-supportive attitudes in the current study were associated negatively with reliance on affective information and positively with reliance on attractiveness information, both at a moderate level (*d*s = −0.56, 0.46). In contrast, but also consistent with prior work, rape-supportive attitudes showed a weak positive association with reliance on clothing style that was not statistically significant (*d* = 0.15). New to the current work, those endorsing more rape-supportive attitudes showed a weak, non-significant tendency toward increased reliance on contextual sexual relevance when judging women’s momentary sexual interest (*d* = 0.22). Overall, these findings are consistent with theoretical perspectives suggesting that sexual-perception processes may increase risk for sexually coercive and aggressive behavior (Abbey et al., 2011; Farris et al., 2008b). In particular, reduced focus on women’s affect and elevated focus on women’s attractiveness—that is, possibly confusing one’s own feelings of sexual interest with a potential partner’s feelings of sexual interest (Maner et al., 2005, 2012)—appear to be problematic. Fortunately, trial-by-trial feedback strongly enhanced affective utilization and moderately reduced attractiveness utilization when judging the sexual interest of decontextualized women, even among higher-risk men who endorsed rape-supportive attitudes 1 SD above the mean (Treat et al., in press).

The mixed-effects modeling approach adopted in the current work estimated a variance-covariance matrix for participant-specific random effects, including the cue-utilization coefficients for the four stimulus dimensions. These pairwise correlations (i.e., standardized covariances) between utilization coefficients indicated to what extent participants utilized the cues independently of one another. Three primary findings emerged. First, men’s reliance on women’s affect and attractiveness showed a markedly strong negative association (*r* = −0.73), suggesting that men focus either on women’s nonverbal affective cues or on women’s attractiveness when judging their momentary sexual interest, but not on both. Second, men’s utilization of women’s clothing style and the sexual relevance of the context converged strongly (*r* = 0.49), indicating that men who rely on clothing style also tend to rely on contextual sexual relevance when judging women’s sexual interest. Notably, both of these cues tend to be more environmental in nature—that is, they are less intrinsic to the woman than affect and attractiveness. Third, the remaining four bivariate correlations between cue utilization coefficients were all small in magnitude.

Inspection of the variance-covariance matrix for random participant-specific effects suggests that the underlying structure of men’s perceptions of women’s nonverbal, dating-relevant cues may be two-dimensional, rather than four-dimensional, as would be expected if participants relied completely independently on the four stimulus dimensions. Indeed, a principal components analysis of the model-estimated utilization coefficients for the four dimensions extracted two components that accounted for 86.5 % of the variability in the data (eigenvalues (λs) = 1.87, 1.59). Attractiveness and affect utilizations correlated 0.94 and −0.92 with the first component, whereas clothing style and contextual sexual relevance utilizations correlated 0.90 and 0.88 with the second component, and all cross-loading correlations were less than |0.30|. It is interesting that the relations between rape-supportive attitudes and cue utilization that have emerged across studies are stronger for affect and attractiveness than for clothing style and for contextual sexual relevance (Treat et al., in press, under review). Pending replication of this potential cue-utilization structure, risk for exhibiting sexually coercive and aggressive behavior may be linked more strongly to the first than the second component. If so, then the conflation of utilization of affect and attractiveness information might prove to be another fruitful target for cognitive-training programs.

To enhance our understanding of participants’ awareness of their cue-utilization patterns, we asked participants to estimate their own cue utilizations after they completed the sexual-interest judgment task. Consistent with observed cue-utilization estimates, participants on average reported relying very strongly on affect, strongly on attractiveness, and to a somewhat lesser degree on both clothing style and contextual sexual relevance. Self-reported estimates also converged strongly with observed estimates for affect and attractiveness, in particular. Finally, endorsement of rape-supportive attitudes correlated negatively with reported reliance on affect and positively with reported reliance on attractiveness, consistent with the observed data. Overall, therefore, college men showed substantial insight into their cue-utilization patterns, consistent with our prior findings (Treat et al., under review), although their awareness may have been bolstered by observing themselves make 173 sexual-interest judgments prior to providing their estimates.

Evidence of significant insight into cue utilization suggests that explicitly instructing men to increase their reliance on affective cues and decrease their reliance on less valid cues like attractiveness and clothing style when judging women’s momentary sexual interest might prove particularly useful for the modification of cue utilization, particularly for affect and attractiveness. Surprisingly, however, explicit instruction produced much weaker effects than trial-by-trial feedback on both affect utilization (*d*s = 0.37, 1.72) and attractiveness utilization (*d*s = −0.33, −0.65; Treat et al., in press, under review). More comprehensive explicit instructions might exert a stronger effect, of course, but it also is possible that insight is insufficient for significant cognitive change in the absence of practice with feedback. Future research profitably might evaluate whether explicit instruction potentiates the effect of trial-by-trial feedback on individual judgments, as in prior work (Treat et al., in press). Alternatively, block-by-block feedback might be provided on participants’ actual cue utilization to enhance participant awareness of their cue-utilization patterns. Such “cognitive/process feedback” (Karelaia & Hogarth, 2008) might prove particularly useful when combined with explicit instruction about the four cues and their differential validity as potential indicators of a woman’s momentary sexual interest.

## Conclusions

College men judging women’s current sexual interest rely not only on nonverbal information in women’s facial expressions and body language but also on the perceived provocativeness of women's clothing and women’s normative attractiveness. Independently of these more woman-specific influences, men also rely on the sexual relevance of the environmental context to draw inferences about women’s momentary sexual interest. Reliance on non-affective indicators of sexual interest may set the stage for unwanted advances or even sexually coercive or aggressive behavior, particularly among men at greater risk of such behavior, who rely less than their peers on affective cues and more than their peers on attractiveness. Across men, usage of attractiveness cues is strongly associated with neglect of affective cues, but use of these two cues is relatively independent of the use of cues of dress or environmental context, suggesting that an underlying structure may govern utilization of these cues. These findings underscore the potential nomothetic and idiographic utility of developing and evaluating comprehensive cognitive-training programs that are designed to enhance college men’s relative focus on women’s affect versus attractiveness and to reduce their focus on the more environmental characteristics of women’s clothing style and contextual sexual relevance. Ultimately, such training programs might prove useful as an adjunct to prevention programs for sexual aggression on college campuses, where existing approaches to this serious behavioral-health problem have proven to be inadequate (e.g., Anderson & Whiston, 2005; DeGue et al., 2014).
